# Supplementary material for: Content-rich biological network constructed by mining PubMed abstracts
Source: BMC Bioinformatics. 2004 Oct 8;5:147. doi: 10.1186/1471-2105-5-147 (PMC528731; doi:10.1186/1471-2105-5-147)
Supplement: Additional File 5 — The original Chilibot query results of the term "long-term potentiation (LTP)" and 22 other terms, limiting the latest references analyzed to the years 1990, 1995, 2000, and 2004. [file 1471-2105-5-147-S5.bz2 › chilibotAdditionalFile5/ltp2000/html/LTP_CAMKII.html]

 


 **LTP** and **CAMKII** 
  
Found 70 abstracts in PubMed,  **30 abstracts were retrieved and analyzed**.  


---

 Search Google  |
 PDF files only 
|  EDU domain only 

---

**Interactive relationship** (e.g. stimulation, inhibition, etc)

- **LTP**  is differentially sensitive to  **CaMKII**  and PKA inhibitors depending on the history of the synapse.  Ref: 10879537 Nature, 2000
- The amount of  **LTP**  observed in alpha  **CaMKII**  mutants was normally distributed, blocked by APV 2.7%, n = 8, and did not correlate with age.  Ref: 10454359 Learn MemLearn Mem, 2000
- Our results suggest that AC activation facilitates  **LTP**  induction by suppressing protein phosphatases and enabling a persistent increase in the levels of autophosphorylated  **CaMKII** .  Ref: 10087064 J Neurosci, 1999
- **CaMKII**  dependent phosphorylation of NR2A and NR2B is decreased in animals characterized by hippocampal damage and impaired  **LTP** .  Ref: 9987018 Eur J Neurosci, 1999
- Here a model of calmodulin binding and trapping by  **CaMKII**  in a dendritic spine was used to estimate levels and durations of  **CaMKII**  activation following  **LTP**  inducing tetani.  Ref: 10798500 J Comput NeurosciJ Comput Neurosci, 1999
- Conversely,  **LTP**  induction in naive synapses and depressed synapses increases phosphorylation of the  **CaMKII**  site and the PKA site, respectively.  Ref: 10879537 Nature, 2000
- Activation of calcium calmodulin dependent protein kinase II  [ **CAMKII** ]   **CaMKII**  by calmodulin following calcium entry into the cell is important for long term potentiation  [ **LTP** ]   **LTP** .  Ref: 10798500 J Comput NeurosciJ Comput Neurosci, 2000
- It is demonstrated numerically that 1  **CaMKII**  is tuned to be activated by stimulation protocols associated with the induction of long term potentiation  [ **LTP** ] .  Ref: 9618904 Comput Chem, 1998
- We recently reported that  **CaMKII**  dependent phosphorylation of the neurofilament L NF L head domain was induced in the apical dendrites during long term potentiation  [ **LTP** ] .  Ref: 10976954 Neuroreport, 2000
- Usually  **LTP**  depends on calcium influx through postsynaptic N methyl D aspartate NMDA type glutamate receptors and subsequent activation of calcium calmodulin dependent protein kinase II  **CaMKII** .  Ref: 10077668 Proc Natl Acad Sci U S A, 1999
- calcium transients that switch  **CaMKII**  from the low phosphorylated state to the high phosphorylated one are in the same range of amplitudes and frequencies as the calcium transients that induce  **LTP** .  Ref: 11053103 Biophys J, 2000
- Activation of the calcium and calmodulin dependent protein kinase II  **CaMKII**  and its conversion into a persistently activated form by autophosphorylation are thought to be crucial events underlying the induction of long term potentiation  [ **LTP** ]   **LTP**  by increases in postsynaptic calcium.  Ref: 10087064 J Neurosci, 1999
- If the total concentration of  **CaMKII**  subunits is significantly higher than the phosphatase Michaelis constant, two stable steady states of the  **CaMKII**  autophosphorylation can exist in a calcium concentration range from below the resting value of the intracellular calcium to the threshold concentration for induction of long term potentiation  [ **LTP** ]   **LTP** .  Ref: 11053103 Biophys J, 2000
- Driving AMPA receptors into synapses by  **LTP**  and  **CaMKII**  requirement for GluR1 and PDZ domain interaction.  Ref: 10731148 Science, 2000
- The calcium calmodulin dependent kinase II  **CaMKII**  is required for hippocampal long term potentiation  [ **LTP** ]   **LTP**  and spatial learning.  Ref: 9452388 Science, 1998
- Furthermore, Ser 57 phosphorylation during  **LTP**  is suggested to be mediated by  **CaMKII** .  Ref: 10854283 J Neurochem, 2000
- CA1 long term potentiation  [ **LTP** ]  is diminished but present in hippocampal slices from alpha  **CaMKII**  mutant mice.  Ref: 10454359 Learn MemLearn Mem, 2000
- Follow up studies on subsequent generations of these mutant mice in a novel inbred background by our laboratories have shown that whereas a deficiency in CA1  **LTP**  is still present in alpha  **CaMKII**  mutant mice, it is different both quantitatively and qualitatively from the deficiency first described.  Ref: 10454359 Learn MemLearn Mem, 2000
- Identification of the postsynaptic adapter for  **CaMKII**  fills a critical gap in the understanding of  **LTP**  because  **CaMKII**  mediated phosphorylation of AMPA receptors is an important step during  **LTP** .  Ref: 10077668 Proc Natl Acad Sci U S A, 1999
- Previous work has shown that mice missing the alpha isoform of calcium calmodulin dependent protein kinase II  [ **CAMKII** ]  alpha  **CaMKII**  have a deficiency in CA1 hippocampal long term potentiation  [ **LTP** ]   **LTP** .  Ref: 10454359 Learn MemLearn Mem, 1999
- calcium calmodulin dependent protein kinase II  **CaMKII**  is a serine threonine protein kinase that regulates long term potentiation  [ **LTP** ]  and other forms of neuronal plasticity.  Ref: 9768845 Neuron, 1998
- Although  **LTP**  at 60 min post tetanus was clearly deficient in these alpha  **CaMKII**  mice 42.6%, n = 33 compared with alpha  **CaMKII**  control animals 81.7%, n = 17, alpha  **CaMKII**  mutant mice did show a significant level of  **LTP** .  Ref: 10454359 Learn MemLearn Mem, 1998
- alpha  **CaMKII**  mRNA, which is localized in dendrites and is necessary for synaptic plasticity and  **LTP** , contains two CPEs.  Ref: 9856468 Neuron, 1998
- In addition, a treatment able to restore long term potentiation  [ **LTP** ]   **LTP**  in hippocampal slices from MAM rats, e.g. exposure to D serine, is able to restore  **CaMKII**  activity to the control value.  Ref: 9987018 Eur J Neurosci, 1999
- Long term potentiation  [ **LTP** ]   **LTP**  at the Schaffer collateral CA1 synapse involves interacting signaling components, including calcium calcium calmodulin dependent protein kinase II  **CaMKII**  and cyclic adenosine monophosphate cAMP pathways.  Ref: 9632393 Science, 1998
- Although this supports a role for alpha  **CaMKII**  in CA1  **LTP** , it also suggests that a form of alpha  **CaMKII**  independent  **LTP**  is present in mice that could be dependent on another kinase, such as the beta isoform of  **CaMKII** .  Ref: 10454359 Learn MemLearn Mem, 1998
- Calcium calmodulin dependent protein kinase II  [ **CAMKII** ]  CaM KII is thought to be involved in the majority of the neuronal functions mediated by intracellular free calcium, and has been implicated in long term potentiation  [ **LTP** ] , learning, and memory.  Ref: 10403830 Biochem Biophys Res Commun, 1999

**Parallel relationship** (e.g. studied together, co-existance, homology, etc.)

- PP1 inhibition seems to promote the activation of calcium calmodulin dependent protein kinase  **CaMKII** , because 1 a  **CaMKII**  inhibitor blocked the induction of  **LTP**  by TPS paired with either isoproterenol or activated inhibitor 1 and 2  **CaMKII**  in area CA1 was activated by the combination of TPS and 8 Br cAMP but not by either stimulus alone.  Ref: 11050107 J Neurosci, 2000
- A significant difference in input output curves was also observed between alpha  **CaMKII**  and alpha  **CaMKII**  animals, suggesting that differences in synaptic transmission may be contributing to the  **LTP**  deficit in mutant mice.  Ref: 10454359 Learn MemLearn Mem, 2000
- Gating of  **CaMKII**  by cAMP regulated protein phosphatase activity during  **LTP** .  Ref: 9632393 Science, 1998
- Site specific phosphorylation of neurofilament L is mediated by calcium calmodulin dependent protein kinase II  [ **CAMKII** ]  in the apical dendrites during long term potentiation  [ **LTP** ] .  Ref: 10854283 J Neurochem, 2000
- These data reveal unexpected complexities in the involvement of KN 62 sensitive processes presumably  **CaMKII**  in the induction of  **LTP** .  Ref: 9704994 NeuropharmacologyNeuropharmacology, 2000
- The blockade of  **LTP**  by a  **CaMKII**  inhibitor was not overcome by thiophosphorylated inhibitor 1.  Ref: 9632393 Science, 1998
- Because increases in calcium can also activate protein phosphatases that oppose persistent  **CaMKII**  activation,  **LTP**  induction may also require activation of signaling pathways that suppress protein phosphatase activation.  Ref: 10087064 J Neurosci, 1999
- These results indicate that the cAMP pathway enables TPS induced  **LTP**  by inhibiting PP1, thereby enhancing calcium independent  **CaMKII**  activity.  Ref: 11050107 J Neurosci, 2000
- These results show that  **LTP**  and  **CaMKII**  activity drive AMPA Rs to synapses by a mechanism that requires the association between GluR1 and a PDZ domain protein.  Ref: 10731148 Science, 2000
- Here we show that NF L is phosphorylated by  **CaMKII**  in a subpopulation of apical dendrites during  **LTP** , indicating that Ser 57 is a novel phosphorylation site of NF L in vivo related to the neuronal signal transduction.  Ref: 10854283 J Neurochem, 2000
- Long term potentiation  [ **LTP** ]   **LTP**  or increased activity of the calcium calmodulin dependent protein kinase II  [ **CAMKII** ]   **CaMKII**  induced delivery of tagged AMPA Rs into synapses.  Ref: 10731148 Science, 2000
- For a molecular understanding of  **LTP**  it is crucial to know how  **CaMKII**  is localized to its postsynaptic targets because protein kinases often are targeted to their substrates by adapter proteins.  Ref: 10077668 Proc Natl Acad Sci U S A, 1999
- These data support the hypothesis that  **CaMKII**  increases postsynaptic responsiveness by increasing the available number of active alpha amino 3 hydroxy 5 methylisoxazole 4 propionic acid kainate channels and suggests that a similar process may occur during the expression of long term potentiation  [ **LTP** ] .  Ref: 9356418 J Neurophysiol, 1997
- Activation and Thr286 autophosphorylation of calcium calmodulindependent kinase II  **CaMKII**  following calcium influx via N methyl D aspartate NMDA type glutamate receptors is essential for hippocampal long term potentiation  [ **LTP** ]   **LTP** , a widely investigated cellular model of learning and memory.  Ref: 9694809 J Biol Chem, 1998
- Thus, the cAMP pathway uses PP1 to gate  **CaMKII**  signaling in  **LTP** .  Ref: 9632393 Science, 1998
